# Composite Tissue Transplantation: A New Era in Transplantation Surgery

**Published:** 2010-09-15

**Authors:** Bohdan Pomahac, Pejman Aflaki

**Affiliations:** Division of Plastic Surgery, Brigham and Women's Hospital, Harvard Medical School, Boston, Massachusetts

## Abstract

Once belonging to science fiction, face transplantation is a clinical reality now. The controversial first face transplantation in Amiens, France in 2005 was followed by significant ethical debate among physicians, surgeons, scientists and bioethicists. The controversies are being followed by cautious optimism and acceptance. An increasing number of surgical teams are developing protocols of composite tissue transplantation. Twenty five years after the birth of the American Society of Reconstructive Microsurgery (ASRM) and during its 25th anniversary, the founding meeting of American Society of Reconstructive Transplantation (ASRT) took place in 2009 to officially introduce the birth of a new field that combines the two disciplines: transplantation and reconstructive microsurgery. It appears that the field of composite tissue allotransplantation will continue to grow for at least several years before more sophisticated means of replacing the damaged tissues become available. This article takes a brief look at the evolution of organ transplantation and reflects our viewpoint regarding the emerging field of composite tissue allotransplantation (CTA).

*Early in the application of any new technology whether it be space travel or heart transplantation, you cannot rely on statistics because there is not enough experience to provide a basis for statistical analysis. As new treatments arise, that special problem of insufficient numbers inevitably arrives for each…. Flexibility and individual judgment must prevail for many years in any new field*.

Francis D. Moore, MD^1^

History of organ transplantation is quite remarkable. Perhaps no other surgical field started with an outlook of significant constraints represented by limited donor pool, or as a temporary solution to medical problems while awaiting more sophisticated means of tissue replacement, and deep ethical challenges. Against the odds and despite being one of the most controversial fields of surgery, transplantation has been one of the fastest growing surgical specialties since its introduction in the mid-20th century. The first organ transplantation by Dr Joseph E. Murray in 1954 was viewed as a rather unique option for identical siblings only, for years, grossly underestimating the historic value of the operation.

The technical expertise in organ transplantation had been based on the pioneering work of Alexis Carrel in early 20th century, who developed a safe and reliable method for vascular anastomosis.^2^ It was not however until the early 1960s and remarkable advances in “microsurgery” that “composite tissue transfer” came into the clinical arena.^3^ The second challenge was to overcome the host immunological barrier, a battle that took almost “seven black years” (1954-1961)^1^ to end. In just more than 7 years after the first successful kidney transplantation between identical twins in 1954,^4^ the advances in transplant immunology and the advent of chemical immunosuppression opened the possibility of transplanting tissues against the genetic barrier leading to the first successful kidney transplant from an unrelated donor in 1962. From the mid-1960s, an increasing number of surgeons, physicians, and scientist began to expand the application of tissue transplantation to other organs. Clinical liver (1963-1966), heart (1968-1973), lung, and pancreas transplantations were important milestones in the evolution of organ transplantation and we feel that we are going through another milestone in the field of transplantation: the rise of the composite tissue allotransplantation (CTA).

In the “hierarchy” of tissue and organ rejection, skin is the most vulnerable organ for transplantation. In fact, the father of immune tolerance Sir Peter Medawar commented on skin as a “non-starter” for a surgeon. The combination of microsurgery and available regimens of chemical immunosuppression led to multiple experiments in CTA with varying degrees of success. Despite the availability of technical expertise, obtaining an efficient yet nontoxic immunosuppressive regimen that could safely prevent rejection of the highly antigenic tissues of multiple embryonic origins, typically including skin, muscles, and nerves, delayed the progress of the field until late 1990s.^5^

The first successful hand transplantation by Professor Dubernard in 1998 was the first clinical application of CTA.^6^ Although revolutionary, this procedure raised remarkable ethical controversies—the use of life-long immune suppressive medications for a non–life-saving tissue. The timing could not have been worse; tissue engineering was going through arguably the most exciting years since its inception and research teams around the world had predicted engineering of human organs within 10 years. In spite of scientific progress and encouraging early predictions, however, tissue engineering proved to be far more challenging and even now appears remote from clinical application.

From 1998 to 2005, only a few centers around the world believed in and performed composite tissue transplantation. It took a second event that led to the greatest excitement about CTA ever. This event was the world's first face transplantation^7^—this time in the setting of discouraging short-term outlook, declining interest, and funding of tissue engineering projects.^8^ Following the initial mistrust, the world's first face transplantation truly excited and energized clinicians around the world. In contrast to conventional “reconstructive” methods, CTA offers the only truly “restorative” procedures for the most disfigured and functionally affected patients. Unlike a prosthesis that may partially return function to an amputated arm, there is no functional prosthesis for replacement of the central facial tissues. The results are remarkable, and these techniques are immediately available for clinicians. Tailored immune-suppression regimens have acceptable rates of complications.

Since the founding meeting of the American Society for Reconstructive Transplantation in July 2008, 2 face transplantation procedures have been performed in the United States.^9^,^10^ Currently, there are approximately a dozen of centers in the United States that are in the process of preparation of their CTA program. Those who were against face transplantation just a few years ago are now admitting that at the current age, it is perhaps the best treatment option in many patients suffering from facial deformities and whom they have treated for many years. At the same time, first reports on transplanted organ tolerance seem to be promising and perhaps achievable in composite tissue transplantation. Simultaneous transplantation of multiple tissues (facial, upper and/or lower extremity) would enhance the quality of life and functional status of polytrauma patients and would appear to further justify life-long immunosuppression for the gained functionality.

We are living in the era of CTA rise. Throughout medical history, there come moments when it is possible to perceive the end of one era and the start of a new one. It is our prediction that until further advances in “tissue engineering” technology are made, the field of composite tissue transplantation continues to grow.

While we must beware the enthusiast who pushes his own operation or his favorite drug too hard, we must equally beware the detached Olympian. The opinion of an inexperienced ethicist may be ethically unacceptable.

Francis D. Moore, MD^1^
